# Whole genome and transcriptome analysis reveal MALDI-TOF MS and SDS-PAGE have limited performance for the detection of the key outer membrane protein in carbapenem-resistant *Klebsiella pneumoniae* isolates

**DOI:** 10.18632/oncotarget.19005

**Published:** 2017-07-05

**Authors:** Naina Adren Pinto, Roshan D’Souza, In Sik Hwang, Jongrak Choi, Yong Ha In, Hyung Soon Park, Choong-Min Ryu, Dongeun Yong, Kyungwon Lee

**Affiliations:** ^1^ Department of Laboratory Medicine and Research Institute of Bacterial Resistance, Yonsei University College of Medicine, Seoul, Korea; ^2^ Brain Korea 21 PLUS Project for Medical Science, Yonsei University, Seoul, Korea; ^3^ ASTA Corporation, Suwon, Korea; ^4^ Molecular Phytobacteriology Laboratory, KRIBB, Daejeon, Korea; ^5^ Biosystems and Bioengineering Program, School of Science, University of Science and Technology, Daejeon, Korea

**Keywords:** MALDI-TOF MS, outer membrane protein, transcriptomic analysis, *Klebsiella pneumoniae*, whole-genome sequencing, Immunology and Microbiology Section, Immune response, Immunity

## Abstract

To detect the outer membrane protein (OMP), which plays a key role in carbapenem resistance, whole-genome and transcriptome analysis of the clinical carbapenem-resistant *Klebsiella pneumoniae* was carried out. The index strain lacked both OmpK35 and OmpK36, whereas the other strains lacked only OmpK35. After SDS-PAGE, the putative OMP bands were excised and identified as OmpA and OmpK36. MALDI-TOF MS showed peaks at ∼36 and ∼38 kDa that corresponded to OmpA and OmpK36, respectively. In all the strains except YMC2014/03/P345, the ∼38 kDa peaks were present. The *K*. *pneumoniae* ATCC 13883 isolate showed three bands on SDS-PAGE and three corresponding peaks on MALDI-TOF MS. The additional third peak at ∼37 kDa corresponding to OmpK35 was observed. To verify OmpK35 peak detection in other *K. pneumoniae* isolates by MALDI-TOF MS, we analyzed six strains from our laboratory’s strain bank. Whole genome sequence indicated that only two isolates had intact OmpK35. Both MALDI-TOF MS and SDS-PAGE did not show a ∼37 kDa peak or an OmpK35 band as observed in the *K. pneumoniae* ATCC 13883 isolate. Separation using SDS-PAGE showed a single peak representing OmpA. Therefore, both SDS-PAGE and MALDI-TOF MS were not completely reliable for OMP detection because they fail to detect OmpK35. To the best of our knowledge, this is the first report on the performance of SDS-PAGE and MALDI-TOF MS for the detection of OMP’s using whole-genome and RNA sequencing analyses.

## INTRODUCTION

Matrix-assisted laser desorption ionization-time of flight mass spectrometry (MALDI-TOF MS) is used for rapid bacterial identification in hospital settings [1], and some studies have validated its use for the detection of outer membrane proteins (OMPs) or porins [2, 3]. Porins are a component of the outer membrane of Gram-negative bacteria that play an important role in the diffusion of bacterial nutrients and antimicrobial agents across the outer membrane [4]. *Klebsiella pneumoniae* is an opportunistic Gram-negative pathogen and a common nosocomial microbe that causes human infection with a mortality rate of 50% [5]. It is worrisome to find increased numbers of carbapenem-resistant *K. pneumoniae* strains because carbapenems are considered the last-resort antibiotics. The resistance mechanisms of carbapenem-resistant *K. pneumoniae* have been attributed to the loss of either one or both of the porins, OmpK35 and OmpK36 [6, 7], in combination with the production of extended-spectrum β-lactamases (ESBLs) or carbapenemase. Although the OmpK35 in *K. pneumoniae* is homologous to OmpF in *Escherichia coli*, the porin channel is significantly larger than that of *E. coli* [8]. Therefore, a lack of OmpK35 increases the multi-drug resistance in *K. pneumoniae*. At present, the detection of porins using sodium dodecyl sulfate-polyacrylamide gel electrophoresis (SDS-PAGE) is widely performed. Even though the process is laborious and time-consuming, the data obtained have been regarded as accurate. However, a recent study indicated discrepancies in the results from *K. pneumoniae*; specifically, the OmpK35 band was not detected by SDS-PAGE, while the corresponding peak was detected using MALDI-TOF MS [3]. It was therefore concluded that MALDI-TOF MS was a better detection method. However, the study was confined to the correlation between SDS-PAGE and MALDI-TOF MS without whole-genome sequence analysis or the examination of protein expression levels. The aim of the present study was to validate the results obtained from SDS-PAGE and MALDI-TOF MS using whole-genome sequencing (WGS) and a transcriptomic analysis of carbapenem-resistant *K. pneumoniae* strains, and to ascertain the reproducibility of MALDI-TOF MS. We also evaluated and compared the performance of MALDI-TOF MS using two different available instruments, the Microflex LT (Bruker Daltonik GmbH, Bremen, Germany) and Tinkerbell LT (ASTA, Suwon, Korea) mass spectrometers.

## RESULTS

**Figure 1:**
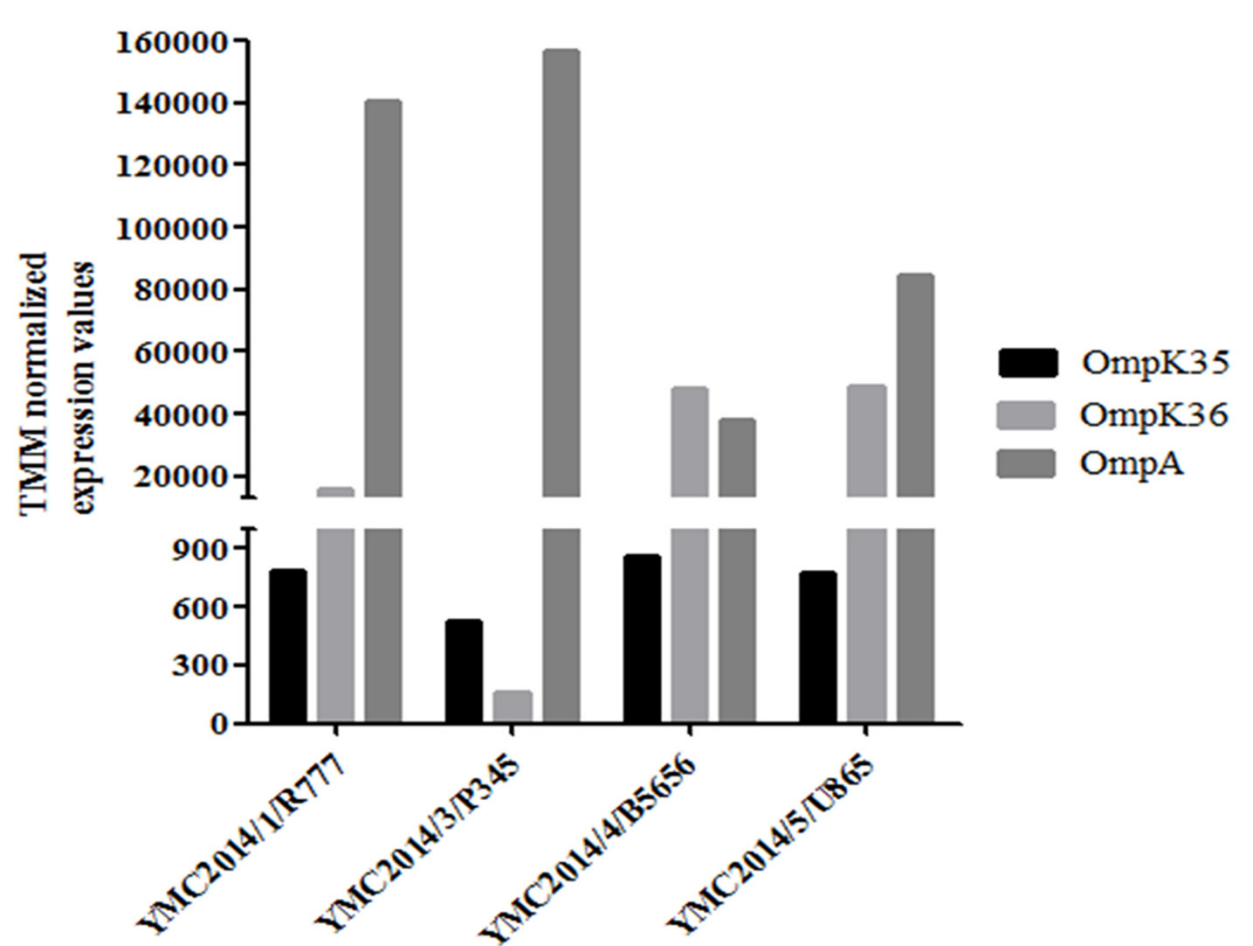
Trimmed mean of M-value-normalized values of the four carbapenem-resistant *Klebsiella pneumoniae* isolates for *OmpA*, *OmpK35*, and *OmpK36* gene expression The actual values are shown in Supplementary Table 2.

Data on MIC, β-lactamase genes, and porins for all isolates are given in Table 1. Additional information for all the isolates, i.e. sex, age, specimen and diagnosis is available in Supplementary Table 1. Sequence analysis of all the four carbapenem-resistant isolates showed that OmpK35 was absent in all isolates due to the deletion of a nucleotide at the 54th position, leading to the early transcriptional termination of the gene. Sequence analysis also indicated that the OmpK36 porin in the strain YMC2014/03/P345 was interrupted by the insertion of a transposon that hindered its expression, whereas no mutations were observed in the other three isolates. While analyzing the RNA data for this study, trimmed mean of M-value (TMM) was used to normalize the transcriptomic gene expression values because the coefficient of variation (CV) was 0.3387, which was lower than the values of 0.342 and 0.3396 for reads per kilobase per million mapped reads (RPKM) and relative log expression (RLE), respectively (Supplementary Table 2). The data for *OmpA*, *OmpK35*, and *OmpK36* in carbapenem-resistant isolates normalized using TMM are shown in Figure 1. The normalized values indicate that *OmpK35* porin expression values were obtained, despite truncation, in all isolates, whereas in YMC2014/03/P345 the *OmpK36* expression was found to be negligible.

**Table 1:** Characteristics of the strains used in this study

Strain	MIC (µg/mL)				β-Lactamase genes present in WGS	Band in MALDI TOF MS/SDS-PAGE				Mutation of porin genes in WGS			References
	IPM		ETP	MEM		36,200 m/z*/ 35.5 kDa*	37,500 m/z**/ 36 kDa**	38,200 m/z***/ 37 kDa***	38,400 m/z / 37.1 kDa	OmpA	OmpK35	OmpK36	
QC Strain													
ATCC 13883	0.5		≤0.5	≤0.25	*bla*_SHV-1_	+/+	+/−	+/+	−/+	NM	NM	NM	This study
Carbapenem-resistant *K. pneumoniae*													
YMC2014/1/R777	1	4		0.5	*bla*_SHV-12_, *bla*_DHA-1_, *bla*_LEN-11_	+/+	−/−	+/+	−/−	NM	54T deletion	NM	This study
YMC2014/3/P345	>32	≥8		≥16	*bla*_SHV-12_, *bla*_DHA-1_, *bla*_LEN-11_	+/+	−/−	−/−	−/−	NM	54T deletion	Transposon insertion	This study
YMC2014/4/B5656	0.75	4		≤0.25	*bla*_SHV-11_, *bla*_DHA-1_	+/+	−/−	+/+	−/−	NM	54T deletion	NM	This study
YMC2014/5/U865	0.5	4		1	*bla*_SHV-11_	+/+	−/−	+/+	−/−	NM	54T deletion	NM	This study
Panel strains of *K. pneumoniae*													
YMC2011/7/B36	1	0.75		0.25	*bla*_SHV-11_, *bla*_SHV-12_, *bla*_DHA-1_,	+/+	−/−	+/+	−/−	NM	54T deletion	NM	[12]
YMC2011/7/B774	0.25	1		0.25	*bla*_OXA-1_, *bla*_CTX-M-15_, *bla*_SHV-11_, *bla*_TEM-1_	+/+	−/−	−/−	−/−	NM	NM	Multiple point mutations	[12]
YMC2013/6/B3993	0.25	0.5		0.25	*bla*_TEM-1_, *bla*_SHV-12_, *bla*_CTX-M-15_, *bla*_OXA-9_	+/+	−/−	+/+	−/−	NM	*IS1* insertion	NM	[13]
YMC2011/8/B10311	0.5	2		2	*bla*_TEM-1_, *bla*_SHV-11_	+/+	−/−	−/−	−/−	NM	*OmpK35_v2* variant	Multiple point mutations	[12]
YMC2011/11/B1440	1	1		1	*bla*_SHV-11_, *bla*_SHV-12_, *bla*_DHA-1_	+/+	−/−	+/+	−/−	NM	54T deletion	NM	[12]
YMC2011/11/B7578	1	4		1	*bla*_SHV-12_, *bla*_DHA-1_, *bla*_SHV-158_	+/+	−/−	−/−	−/−	NM	54T deletion	313G deletion	[12]

WGS, whole-genome analysis; +, present; −, absent; NM, no mutation.

*, OmpA; **, OmpK35; ***, OmpK36

IPM, imipenem; ETP, ertapenem; MEM, meropenem

**Figure 2:**
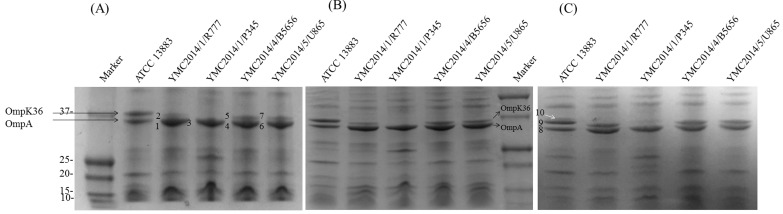
Sodium dodecyl sulfate polyacrylamide gel electrophoresis analysis of outer membrane proteins extracted from four carbapenem-resistant *K. pneumoniae* strains and the carbapenem-susceptible strain ATCC 13883 using various separation gels and growth media (**A**), ExpressPlus™ PAGE 12% (w/v) polyacrylamide gel, isolates grown in Luria-Bertani broth; (**B**), ExpressPlus™ PAGE 4-12% gradient gel, grown in LB broth; (**C**), ExpressPlus™ PAGE 12% (w/v) gel, grown in nutrient broth. The bands numbered 1 to 10 represent the bands excised for liquid chromatography mass spectrometry. Bands 1, 3, 4, 6, and 8 represent OmpA; bands 2, 5, 7, and 9 represent OmpK36; band 10 contains both OmpK35 and OmpK36.

For OMP analysis using SDS-PAGE, the samples were extracted from high-osmolarity LB broth and separated using 12% (w/v) polyacrylamide and gradient gels. The separation pattern was found to be similar in both types of gel (Figures 2a and 2b). We focused on the bands between 30 kDa and 38 kDa, where OmpK35 and OmpK36 are usually present. We found that for the YMC2014/03/P345 strain the OmpK36 band was absent in both gels, whereas the other three carbapenem-resistant isolates showed two discrete bands. In detail, SDS-PAGE showed a prominent band at ∼35.5 kDa in all four resistant strains, which was previously considered to be OmpK35 [2, 9]. Surprisingly, this band was identified to be OmpA using LC-MS/MS (Supplementary Table 3). Thus, all isolates lacked OmpK35 porin expression when analyzed using SDS-PAGE, which is consistent with the WGS data. The extraction of OMPs was also carried out using a low-osmolarity nutrient broth because a high expression of OmpK35 in low-osmolarity media was reported previously [10]. To confirm our findings, samples extracted from low-osmolarity nutrient broth were also run on a 12% (w/v) polyacrylamide gel. It is interesting that the *K. pneumoniae* ATCC 13883 control strain showed an additional band (Figure 2c) above the OmpK36 band that was identified to be a combination of OmpK35 and OmpK36 (Supplementary Table 3). This additional band migrating behind the OmpK36 band might reflect a difference in the migration patterns of porins between strains irrespective of their molecular weight [11]. Six random *K. pneumoniae* isolates (YMC2011/7/B36, YMC2011/7/B774, YMC2013/6/B3993, YMC2011/8/B10311, YMC2011/11/B1440, and YMC2011/11/B7578) were selected from the panel strain bank [12, 13] (Table 1). YMC2013/6/B3993 isolate lacked the *OmpK35* gene due to the insertion of the *IS1* transposon in the gene. OmpK35 was also found to be truncated in YMC2011/7/B36, YMC2011/11/B1440, and YMC2011/11/B7578 due to the deletion of a single nucleotide, i.e. deletion of the nucleotide at the 54th position, similar to the other carbapenem-resistant isolates. Only YMC2011/7/B774 and YMC2011/8/B10311 had intact OmpK35 based on their WGS data. However, the band patterns for all the panel strain isolates on SDS-PAGE did not show any signs of OmpK35 (Supplementary Figure 1). Only a single band representing OmpA was expressed in YMC2011/7/B774, YMC2011/8/B10311, and YMC2011/11/B7578 isolates because they lacked the OmpK36 porin (Table 1). Since no additional band was seen in YMC2011/7/B774 and YMC2011/8/B10311, despite the absence of Coomassie blue staining, empty gels from these isolates were excised at the same height as that of band 10 and identified using LC-MS (data not shown).The proteins were identified to be a mixture of bisphosphate aldolase and OmpA while OmpK35 was completely absent in the excised gel.

**Figure 3:**
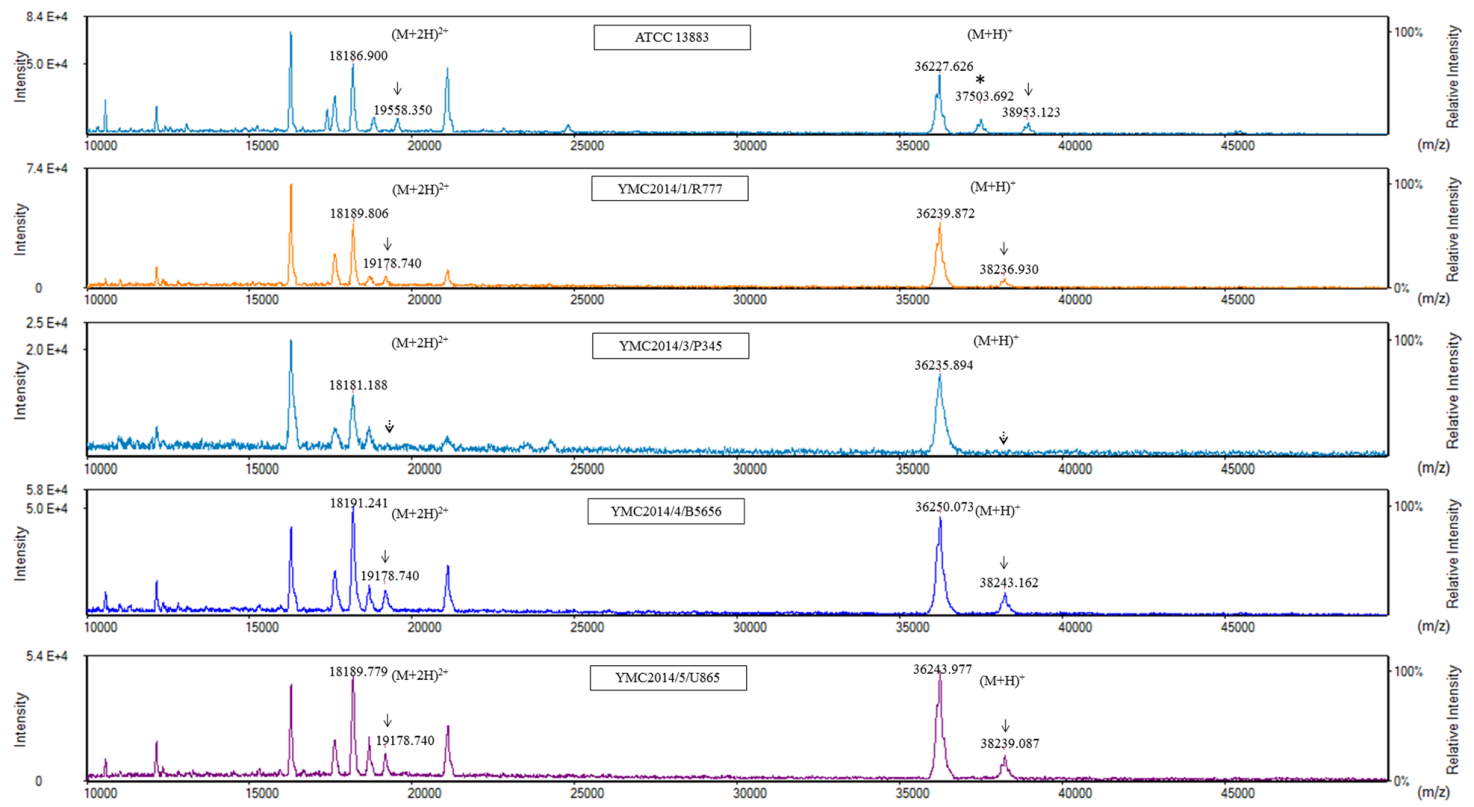
Matrix-assisted laser desorption ionization-time of flight mass spectrometry (MALDI-TOF MS) analysis of carbapenem-resistant *K. pneumoniae* isolates using Tinkerbell LT mass spectrometer (ASTA, Seoul, Korea) The x-axis represents the mass per charge in Daltons (m/z) and the y-axis represents the relative intensity. The 38 kDa peak and its corresponding (M+2H)^2+^ peak at 19 kDa, indicated by solid black arrows, represent OmpK36. The dotted arrows indicate the loss of OmpK36. The peak at 36 kDa indicates OmpA. The asterisk (*) indicates the extra peak corresponding to sodium dodecyl sulfate polyacrylamide gel electrophoresis band 10 consisting of both OmpK35 and OmpK36.

**Figure 4:**
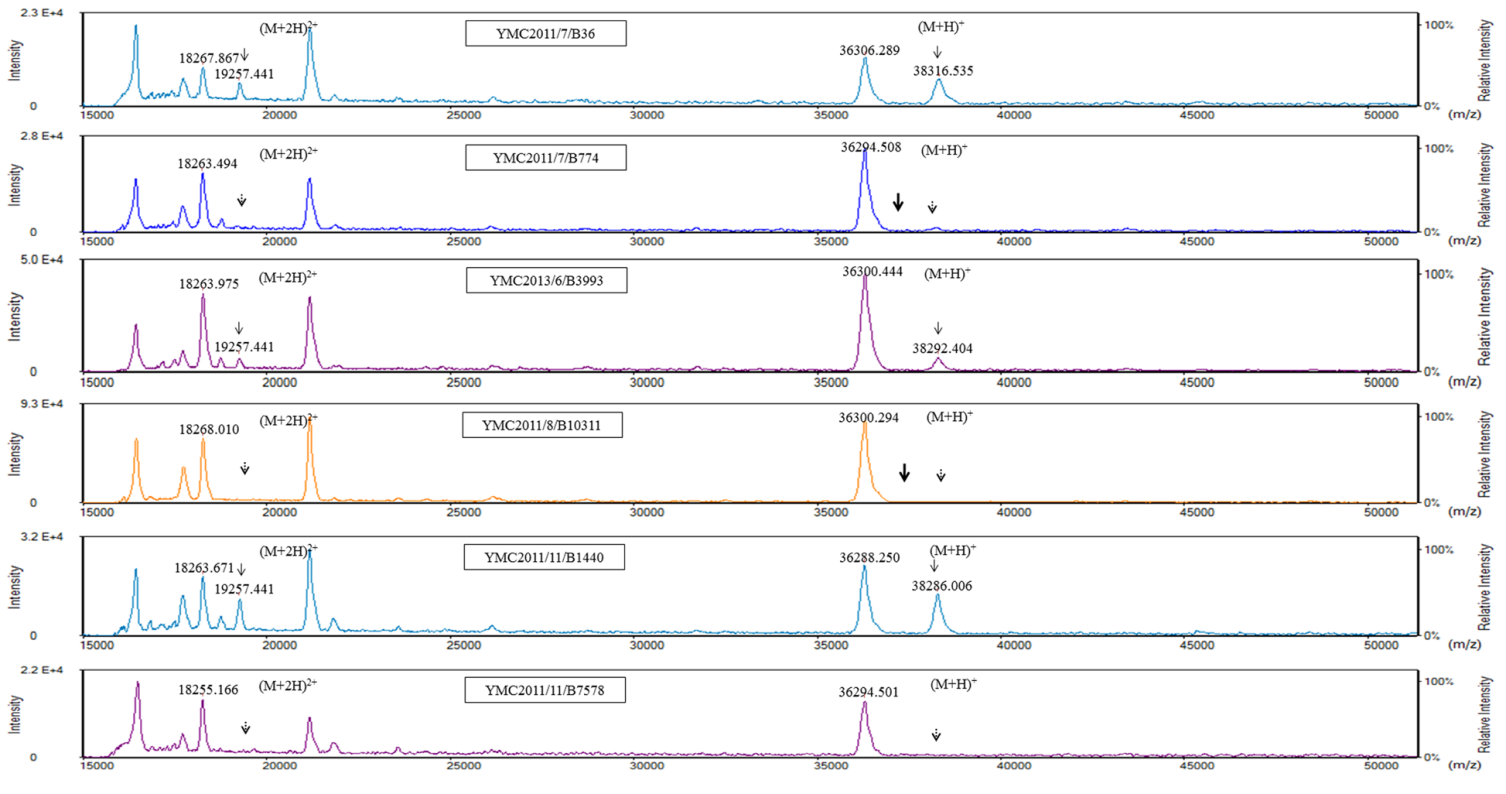
MALDI-TOF MS analysis of panel strains of *K. pneumoniae* using a Tinkerbell LT mass spectrometer The x-axis represents the mass per charge in Daltons (m/z) and the y-axis represents the relative intensity. The 38 kDa peak and its corresponding (M+2H)^2+^ peak at 19 kDa, indicated by solid black arrows, represent OmpK36. The dotted arrows indicate the loss of OmpK36. The absence of the 37 kDa peak is indicated by the bold arrow in isolates YMC2011/7/B774 and YMC2011/8/B10311. Although these isolates carry the *OmpK35* gene, the corresponding peak for the OmpK35 protein was not observed.

For MALDI-TOF MS analysis of the OMPs, the samples extracted from high-osmolarity LB broth did not give reproducible results when analyzed using either the Microflex LT or Tinkerbell LT instrument (data not shown). OMPs extracted from isolates grown in low-osmolarity nutrient broth were further analyzed using both the Tinkerbell LT (Figures 3 and 4) and Microflex LT instruments (Supplementary Figure 2). The data obtained from both the instruments were consistent with each other. Four peaks at ∼18, ∼19, ∼36, and ∼38 kDa were observed on analysis using Tinkerbell LT (Figure 3). The peaks at ∼18 and ∼19 kDa were considered to be multi-charged states of the ∼36 and ∼38 kDa peaks, respectively. We presumed that the ∼36 kDa peak represents OmpK35, while the ∼38 kDa peak represents OmpK36, by reference to a previous study [2]. However, the four carbapenem-resistant isolates lacked OmpK35 and yet had a prominent ∼36 kDa peak. By correlating these data with the peptide sequencing data, we could conclude that the ∼36 kDa peak was in fact OmpA and not OmpK35. Consistent with the findings of WGS and SDS-PAGE, YMC2014/03/P345 lacked the peak at ∼38 kDa, indicating a lack of OmpK36 porin expression. Three peaks were detected for *K. pneumoniae* ATCC 13883 (Figure 3), corresponding to bands 8, 9, and 10 as observed on the SDS-PAGE gel (Figure 2c). The additional ∼37 kDa peak corresponding to band 10 represents the OmpK35 porin. The peak detection using Tinkerbell LT was repeated using the above strains. The data from two additional biological repeats as well as two technical repeats are available in the Supplementary Figure S3-S6. When OMPs of *K. pneumoniae* panel strains were analyzed by MALDI-TOF MS, a prominent ∼36 kDa peak was observed for the strains YMC2013/6/B3993, YMC2011/7/B36, YMC2011/11/B1440, and YMC2011/11/B7578, all of which lack OmpK35 (Figure 4), indicating that the ∼36 kDa peak indeed represented OmpA. It is interesting that the peak corresponding to OmpK35 was not detected in MALDI-TOF MS for the YMC2011/7/B774 and YMC2011/8/B10311 isolates, even though the gene was intact. This led us to conclude that MALDI-TOF MS replicated the results obtained from SDS-PAGE and did not provide any additional data.

The molecular weights of the protein products of *OmpK35, OmpK36* and *OmpA* were calculated (Table 2). The expected molecular weights did not accurately correlate with the peaks obtained from MALDI-TOF MS. The calculated values were the same with respect to OMPs in different isolates except for OmpK35 in YMC2011/8/B10311(variant named *OmpK35_v2*), due to the substitution of leucine to valine at position 14 in the leading peptide [14]. Since both leucine and valine are branched-chain amino acids, the substitution did not affect OmpK35 expression.

**Table 2:** Predicted molecular weights of OMP gene products based on WGS data

Strain	OmpK35 (Da)	OmpK36 (Da)	OmpA (Da)
ATCC 13883	39509.46	40078.05	38975.79
YMC2014/1/R777	-	40078.05	38975.79
YMC2014/3/P345	-	-	38975.79
YMC2014/4/B5656	-	40078.05	38975.79
YMC2014/5/U865	-	40078.05	38975.79
YMC2011/7/B36	-	40078.05	38975.79
YMC2011/7/B774	39495.43	-	38975.79
YMC2013/6/B3993	-	40078.05	38975.79
YMC2011/8/B10311	39509.46	-	38975.79
YMC2011/11/B1440	-	40078.05	38975.79
YMC2011/11/B7578	-	-	38975.79

## DISCUSSION

MALDI-TOF MS is gaining momentum at present because of its rapid identification capability, cost-effectiveness, and reliability. Recently, apart from bacterial identification, a few studies have validated its use for the detection of the OmpK35 and OmpK36 porins in *K. pneumoniae* [2, 3]. However, our current study exposes the limitation of MALDI-TOF MS for the detection of OmpK35. We compared the results of SDS-PAGE, WGS, and RNA sequence analyses to provide sufficient data to validate our claims.

Generally, WGS gives an accurate genetic overview regarding the mutations present in the genes, while transcriptomic analysis provides a better insight into their expression. In this study, both SDS-PAGE and MALDI-TOF MS failed to detect OmpK35 in all clinical isolates, except the *K. pneumoniae* ATCC 13883 strain. Therefore, our data is inconsistent with the previous study [3], where a peak at ∼37 kDa in MALDI-TOF MS was always prominent even though the corresponding bands were absent in SDS-PAGE. The absence of WGS and transcriptomic analysis data in their study limits the scope for further comparison. The presence of the OmpK35 band in *K. pneumoniae* ATCC 13883 may have occurred because of the use of low-osmolarity media, i.e., nutrient broth, for enhancing OmpK35 expression as reported previously [10].

Although WGS detected the presence of intact OmpK35 in YMC2011/7/B774 and YMC2011/8/B10311 isolates without truncation, no bands were present on SDS-PAGE. LC-MS is more sensitive than Coomassie blue staining, and therefore, to identify whether OmpK35 peptides are truly absent, bands were excised from the same height as that of band 10 (Figure 2c) in the YMC2011/7/B774 and YMC2011/8/B10311 isolates. OmpK35 was absent irrespective of its isolation with nutrient broth; instead, the bands were identified to be a mixture of bisphosphate aldolase and OmpA. These proteins identified in empty gels in absence of Coomassie blue staining proves that very low protein concentrations may not undergo effective staining. In addition, no peaks were observed on MALDI-TOF MS for OmpK35 in these two isolates. This finding also indicates that MALDI-TOF MS reproduces the data obtained from SDS-PAGE.

The additional band in ATCC 13883 above OmpK36, which was a mixture of both OmpK35 and OmpK36, gave a single 37 kDa peak on MALDI-TOF MS. We believe that the constitution of the band and its location on the gel did not affect the position of the OmpK35 peak. Hu et al. (2015) described that the peak observed at 37 kDa represented OmpK35, which was not detected on SDS-PAGE. Their study also analyzed *K. pneumoniae* ATCC 13883 and identified a 37 kDa peak and its multi-charged state at 18.5 kDa. However, in our study, we were unable to find the multi-charged state of the 37 kDa peak in *K. pneumoniae* ATCC 13883, nor could we identify both OmpK35 peaks in the YMC2011/7/B774 and YMC2011/8/B10311 isolates. Therefore, we spotted a discrepancy regarding the ability to obtain the OmpK35 peak using MALDI-TOF MS.

In addition, the expected protein molecular weight of the OMPs did not correlate with the MALDI-TOF MS peak obtained (Table 2). This may be due to the direct analysis of extracted OMPs without further purification. The extracted OMP sample contains some amount of salts and other components from the buffer as well as from the steps involved in the extraction procedure. This might suppress the signals from MALDI-TOF MS, thus resulting in a difference in the obtained peaks [15].

The limitation of this study was that the transcriptomic data available in our study was limited to carbapenem-resistant strains, which lacked OmpK35. The normalized values of OmpK35 might represent the expression of truncated proteins. This study also partially illustrates the limitation of using transcriptomic data alone for data interpretation.

From the above analysis, we can reach two conclusions: (i) MALDI-TOF MS replicates SDS-PAGE results for porin detection in *K. pneumoniae*. MALDI-TOF MS and SDS-PAGE show similar results even though they did not correlate with the WGS and transcriptome data; for example, the failure to detect OmpK35. Thus, to save time, MALDI-TOF MS can replace SDS-PAGE. In addition, the results obtained using the Tinkerbell LT instrument were replicable in low-osmolarity broth over several repeats. (ii) Both MALDI-TOF MS and SDS-PAGE failed to detect peaks and bands representing OmpK35 expression. In this regard, the data obtained in this study were inconsistent with those from previous studies. The inability of these methods to detect OmpK35 may be attributed to the detection limits of these two methods. This is the first report of the limitation of MALDI-TOF MS for the detection of OmpK35.

Based on the above conclusions, we stress that although MALDI-TOF MS can replace SDS-PAGE for quicker analysis, neither of these methods can be used for porin detection in carbapenem resistant *K. pneumoniae*. Changes to the OMP extraction process may yield better extracts that can be detected using SDS-PAGE or MALDI-TOF MS. Another alternative could be the use of a specific antibody for OMP identification while analyzing the SDS-PAGE data. Studies that have previously identified the OmpA band as OmpK35 without further confirmation should be re-evaluated.

## MATERIALS AND METHODS

### Bacterial strains and identification

Eleven *K. pneumoniae* strains were used in this study including four carbapenem-resistant strains, the susceptible isolate ATCC 13883, and six other isolates from a panel of strains maintained by our laboratory consisting of both carbapenem-resistant and susceptible strains (Table 1). In this study, the term ‘carbapenem-resistant’ implies that the strain was resistant to at least one of the carbapenems, namely ertapenem, meropenem, or imipenem. The isolates were identified using a Bruker MALDI Biotyper CA System (Bruker Daltonik GmbH).

### Antimicrobial susceptibility testing

The minimum inhibitory concentration (MIC) of carbapenems for all the strains was determined using the agar dilution and E-test methods, which were interpreted as described in the Clinical Laboratory Standards Institute (CLSI) guidelines [16].

### Outer membrane protein extraction and analysis

OMP samples were extracted using both a low-nutrient broth and a high-osmolarity Luria-Bertani (LB) broth as previously described [11]. The obtained OMP sample was heated for 5 min at 100°C and placed on ice immediately. Extracted samples were separated using SDS-PAGE at a constant voltage of 60V for about 3 h in both a 12% (w/v) polyacrylamide gel and a gradient gel (ExpressPlus™ PAGE Gel, GenScript, Piscataway, NJ, USA). The bands were detected using Coomassie brilliant blue R-250 staining.

### OMP detection using MALDI-TOF MS

The OMP samples extracted for SDS-PAGE were also used for detection by MALDI-TOF MS. The matrix was 40 mg/ml dihydroxybenzoic acid with 4.44 mg/ml 2-hydroxy-5-methoxybenzoic acid (9:1, w/w) in TA30 buffer (30:70 [v/v] acetonitrile : trifluoroacetic acid 0.1% in water). The samples were analyzed using both Microflex LT (Bruker Daltonik GmbH) and Tinkerbell LT (ASTA) mass spectrometers.

For analysis using the Microflex LT instrument, the sample was diluted by a factor of 10 before the addition of the matrix. The diluted sample was then mixed with the matrix at a ratio of 1:1, and 1µl of the mixture was applied to the plate and air dried. The parameters for the Microflex LT analysis were as follows: mode, linear positive mode 10-50 kDa; ion source voltage 1, 20 kV; ion source voltage 2, 18 kV; lens voltage, 5 kV; linear detector voltage, 2.85 kV; pulsed ion extraction delay, 250 ns; digitizer trigger level, 5 mV; laser beam attenuation, 1.852; laser range, 70%; laser offset, 15%; sample rate, 2ns; electronic gain, 100mV; laser beam focus, −1; laser frequency, 60Hz; number of shots, 500. Protein calibration standard I was used for calibration with a regulated calibration error of 67.5 ppm. The peaks were analyzed using flexAnalysis 3.4 (build 57) software (Bruker Daltonik GmbH).

For analysis using the Tinkerbell LT instrument, the undiluted sample was mixed with the matrix at the same ratio, and 2µl of the mixture was applied to the plate and air dried. The following conditions were maintained for the Tinkerbell LT analysis: range 15-50 kDa, laser power 100%, shots 80×80, locus pattern, edge bias, radius 1,100μm, delay time 3030, and smoothing of 13 points with baseline subtraction for peak processing. An extraction voltage of 18kV and a voltage gradient of 94.7% (17.05 kV) were maintained. Bovine serum albumin protein standards were used for calibration i.e. 33 and 22 kDa representing [M+2H]^+^and [M+3H]^+^, respectively. The analysis using Tinkerbell LT was performed using three biological replicates and two technical replicates.

### Peptide analysis using liquid chromatography tandem mass spectrometry (LC-MS/MS) and database searching

The putative OmpK35, OmpK36, and empty gel bands (at the height of band 10) were excised from the SDS-PAGE gel for peptide analysis. The analysis was performed as previously described [17] by the Yonsei Proteome Research Center, Seoul, South Korea. A nano high performance liquid chromatography system (Agilent, Santa Clara, CA, USA) was used for nano LC-MS/MS analysis. Peptide separation was carried out using a nano chip column. Product ion spectra were analyzed using an Agilent 6530 Accurate-Mass Q-TOF instrument.

The MASCOT algorithm (Matrix Science, London, UK) was used for database searching to identify protein sequences. The criteria used were, taxonomy; *Proteobacteria* (NCBInr downloaded 2015.01.23), OmpK35 (accession no. ADG27468), OmpK36 (accession no. YP_005228001); fixed modification: carbamidomethylated at cysteine residues; variable modification: oxidized at methionine residues; maximum allowed missed cleavage: 2; MS tolerance: 100 ppm; MS/MS tolerance: 0.1 Da. Only peptides obtained from trypsin digestion were considered.

### WGS and data analysis

The Wizard^®^ genomic DNA purification kit (Promega, Madison, WI) was used for DNA extraction according to the manufacturer’s protocol. The Qubit^®^ dsDNA BR assay kit (Molecular Probes, Eugene, OR) was used to estimate the DNA concentration. Library preparation was carried out using an IonXpress™ Plus fragment library kit (Life Technologies, Carlsbad, CA, USA) according to the manufacturer’s protocol. WGS was carried out on a 318 chip v2 using the Ion Torrent PGM™ system and Ion PGM™ Sequencing 400 kit (Life Technologies).

The obtained reads were assembled using the MIRA plug-in (Biomatters, Auckland, New Zealand). The RAST annotation pipeline was used for annotation [18]. Genome analysis was carried out using Geneious 8.1.8 (http://www.geneious.com)(Biomatters). Screening of β-lactamase genes in WGS was carried out using ResFinder (https://cge.cbs.dtu.dk/services/ResFinder/) and further verified using NCBI BLAST. The protein molecular weights of all OMP peptides were calculated using Protein Calculator (https://spin.niddk.nih.gov/clore/Software/A205.html) [19].

### RNA extraction, sequencing, and data analysis

The isolates were grown in high osmolarity LB broth at 37°C till it reached the logarithmic phase and the RNA was extracted using the RNeasy^®^ mini kit (Qiagen GmbH, Hilden, Germany) according to the manufacturer’s protocol. DNA contamination was eliminated using an RNase-free DNase Kit (Invitrogen). The concentration of RNA was measured using a Nanodrop™ spectrophotometer (Thermo Fisher Scientific, Waltham, MA, USA). RNA sequencing was performed using a HiSeq2500 sequencer (Illumina, San Diego, CA, USA) and transcriptomic analysis was carried out using CLRNASeq software (http://www.chunlab.com/software_clrnaseq_download; Chunlab, Seoul, Korea). The data were normalized using the reads per kilobase per million mapped reads (RPKM), relative log expression (RLE), and trimmed mean of M-value (TMM) methods*. K. pneumoniae* ATCC 13883 was used as the reference strain (Assembly ID: GCA_000742135.1).

### Accession numbers

The GenBank accession numbers for the *OmpK35* and *OmpK36* genes from the isolates YMC2014/1/R777, YMC2014/3/P345, YMC2014/4/B5656, and YMC2014/5/U865 are KY019185, KY019186, KY019187, and KY019188 (for *OmpK35*) and KY019189, KY019192, KY019190, KY019191 (for *OmpK36*), respectively.

## SUPPLEMENTARY MATERIALS FIGURES AND TABLES


