# Supplementary material for: Whole genome and transcriptome analysis reveal MALDI-TOF MS and SDS-PAGE have limited performance for the detection of the key outer membrane protein in carbapenem-resistant Klebsiella pneumoniae isolates
Source: Oncotarget. 2017 Jul 5;8(49):84818–26. doi: 10.18632/oncotarget.19005 (PMC5689575; doi:10.18632/oncotarget.19005)
Supplement: Supplementary file 1 [file oncotarget-08-84818-s001.pdf]

Whole genome and transcriptome analysis reveal MALDI-TOF MS and SDS-PAGE have limited performance for the detection of the key outer membrane protein in carbapenem-resistant *Klebsiella pneumoniae* isolates

SUPPLEMENTARY MATERIALS

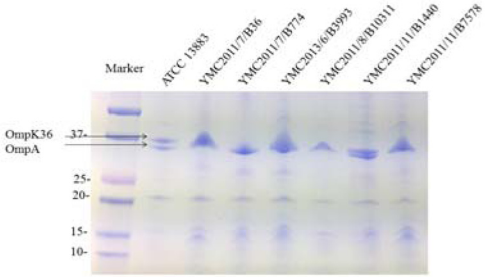

Supplementary Figure 1: SDS-PAGE of 6 panel strains of *k. pneumoniae* isolates. The separation for isolates YMC2011/7/B36, YMC2013/6/b3993, and YMC2011/11/B7578 could not be made sharper even after several attempts with different dilutions of samples.

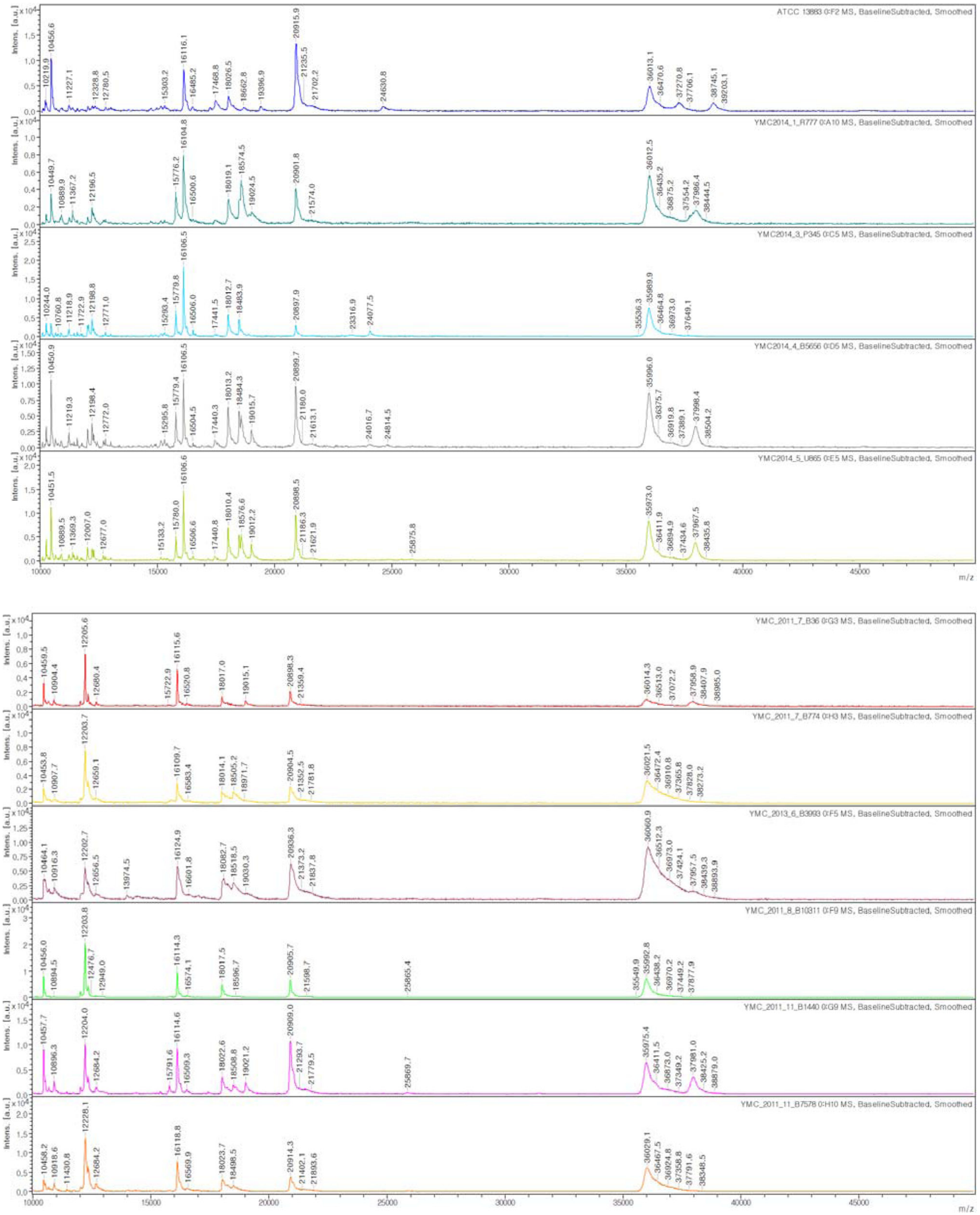

Supplementary Figure 2: MALDI-TOF MS peaks obtained from analysis using a Microflex LT mass spectrometer. The peaks obtained are consistent with those obtained using a Tinkerbell LT mass spectrometer.

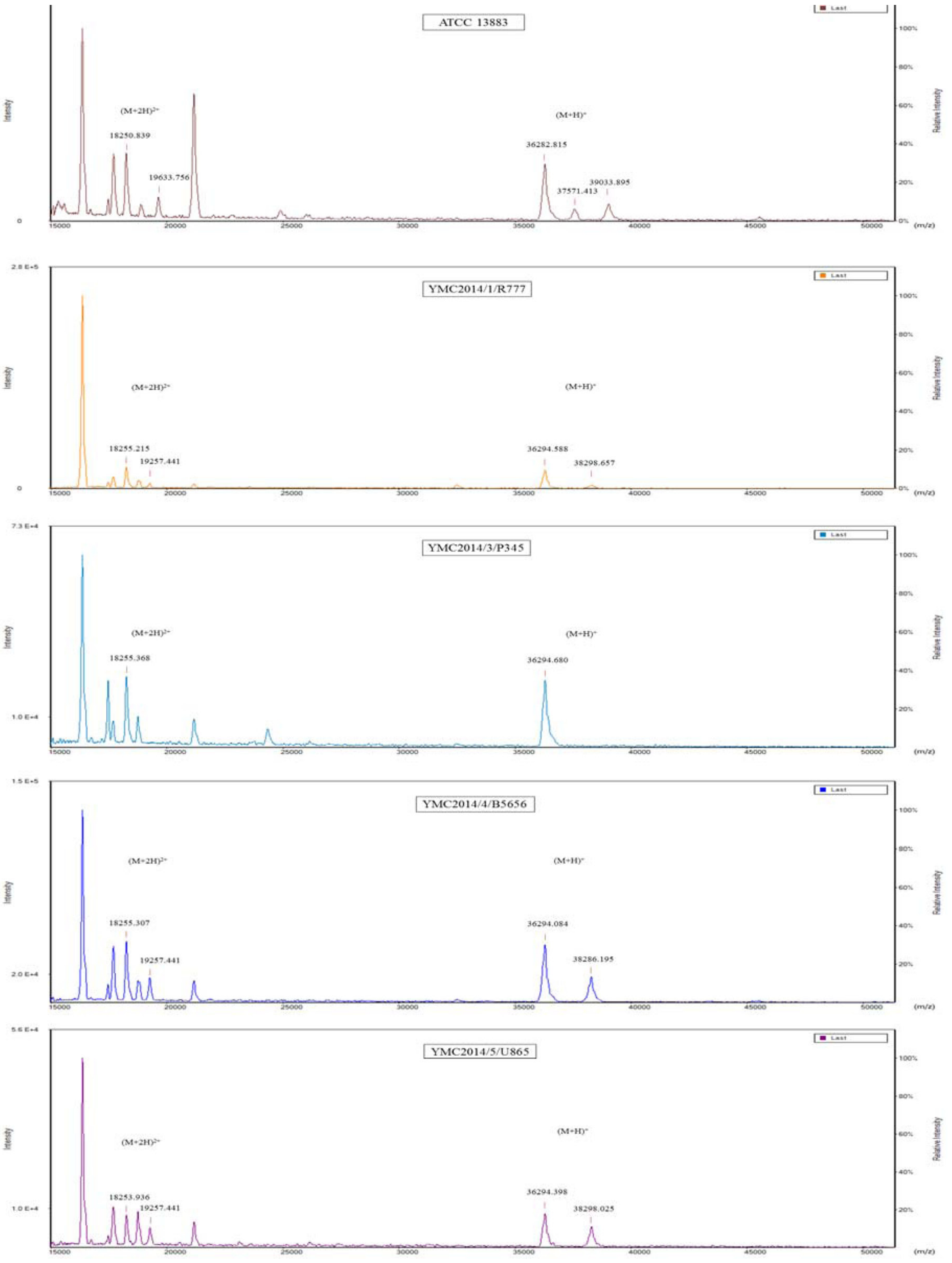

Supplementary Figure 3: Second biological repeat of Figure 3 obtained from the Tinkerbell LT analysis for carbapenem-resistant strains.

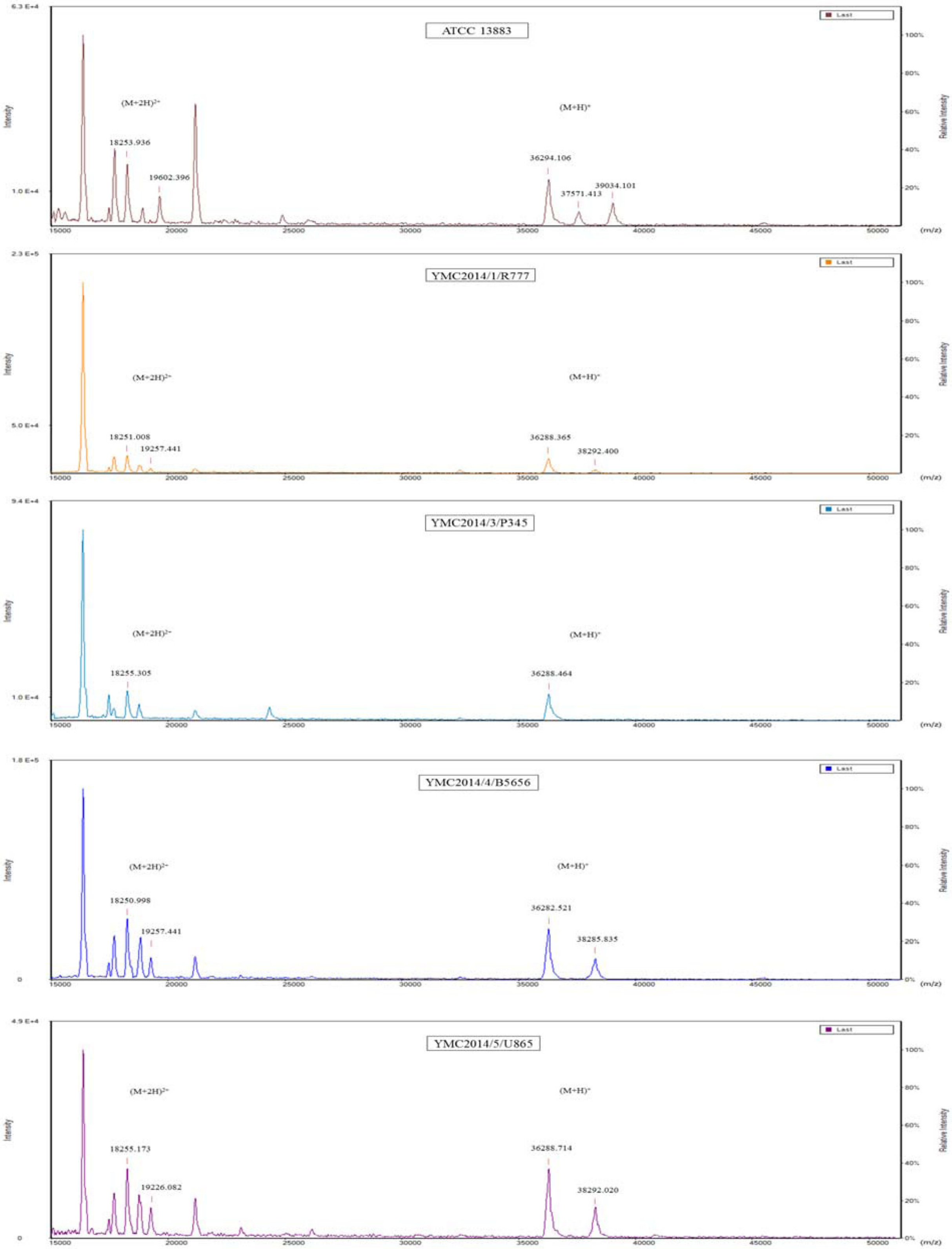

Supplementary Figure 4: Third biological replicate of Figure 3 obtained from the Tinkerbell LT analysis for carbapenem-resistant strains.

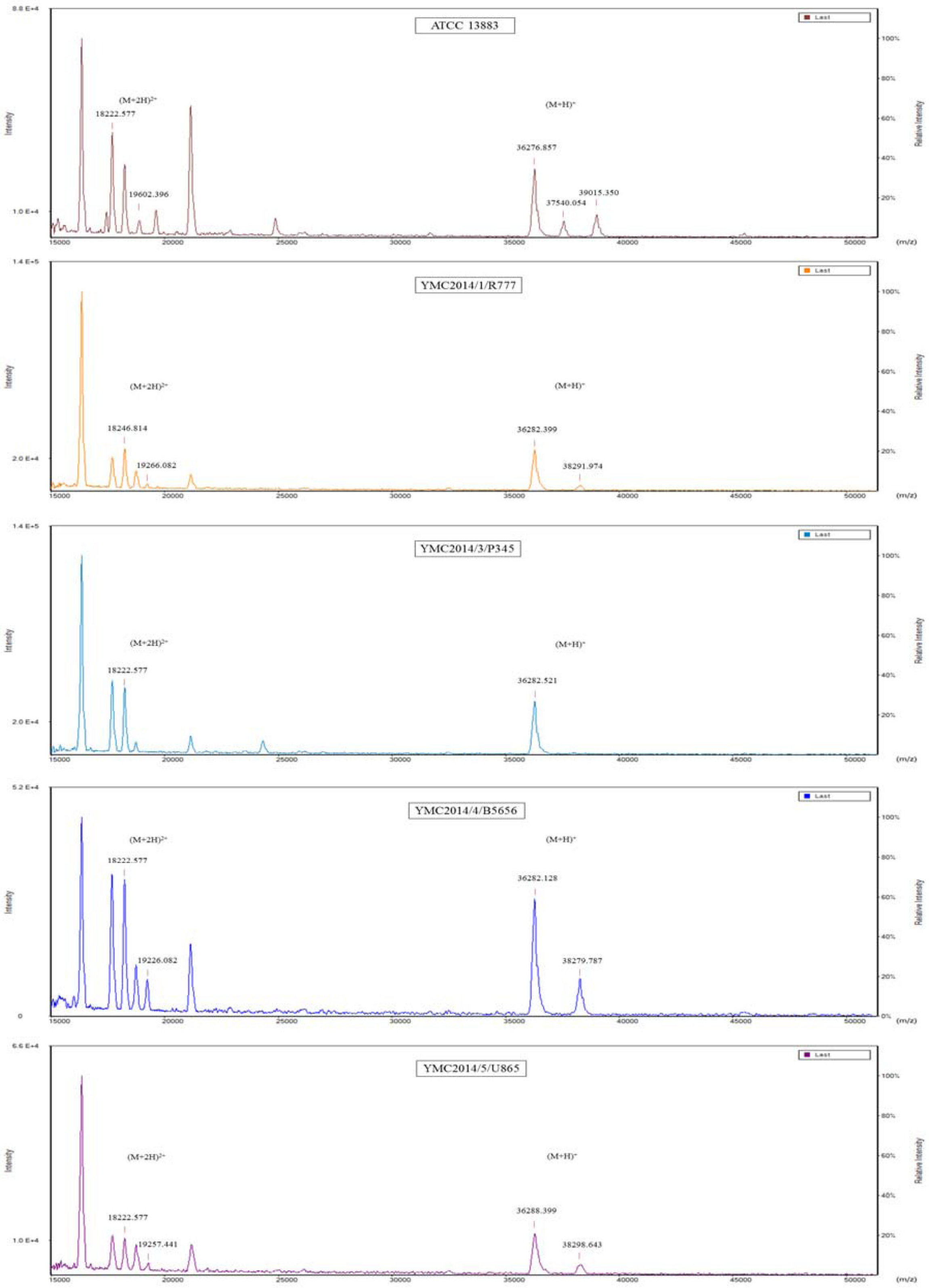

Supplementary Figure 5: First technical repeat of Figure 3 obtained from the Tinkerbell LT analysis for carbapenem-resistant strains.

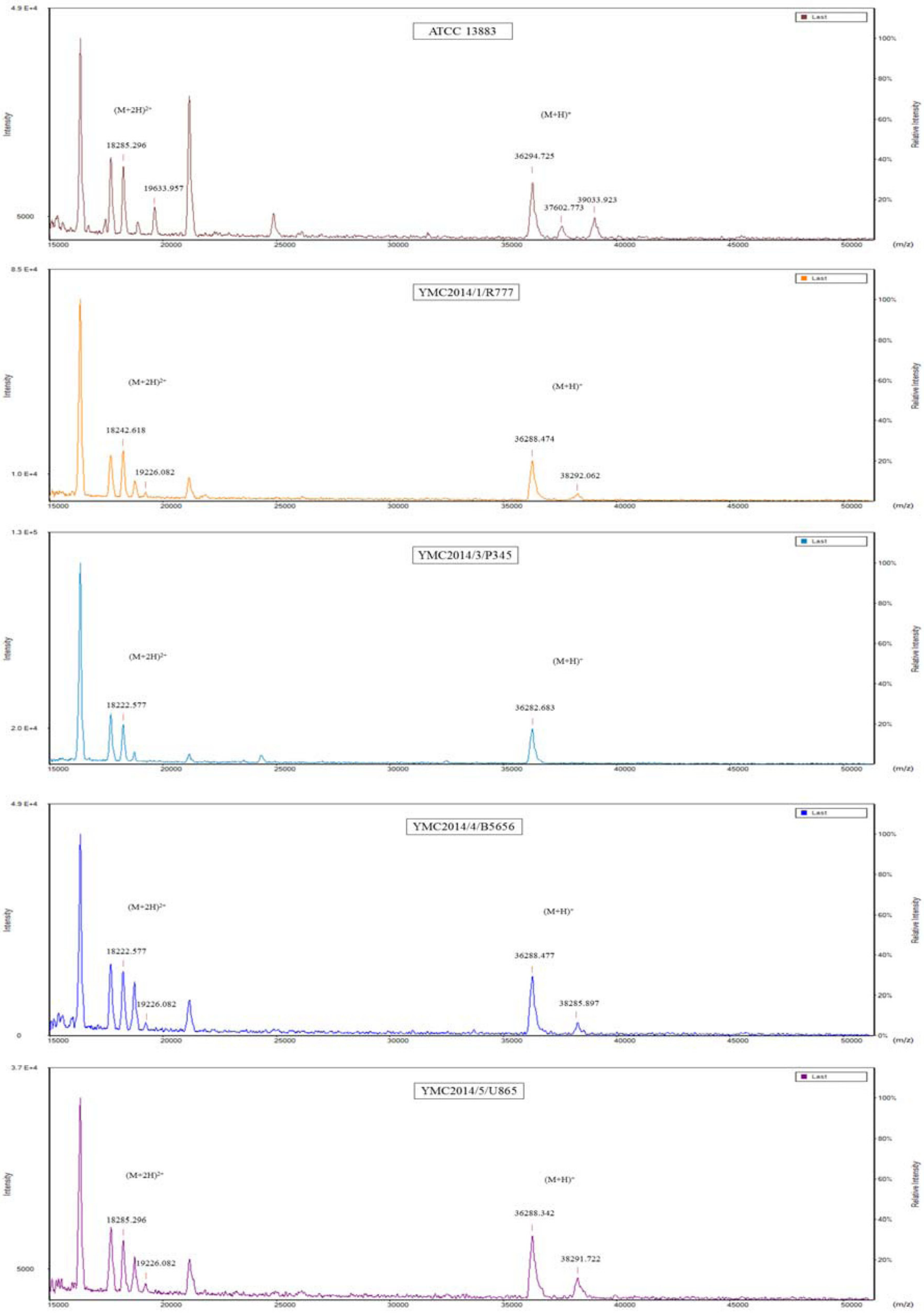

Supplementary Figure 6: Second technical replicate of Figure 3 obtained from the Tinkerbell LT analysis for carbapenem-resistant strains.

Supplementary Table 1: Additional strain details used in the study.

Strain	Sex	Age	Specimen	Diagnosis
YMC2014/1/R777	M	45	Sputum	Infective endocarditis
YMC2014/3/P345	M	45	Pus	Infective endocarditis
YMC2014/4/B5656	M	53	Blood	Leukoencephalopathy
YMC2014/5/U865	M	53	Urine	Leukoencephalopathy
YMC2011/7/B36	M	74	Blood	Multi-drug resistant tuberculosis
YMC2011/7/B774	F	42	Blood	Recurrent cervix cancer
YMC2013/6/B3993	M	70	Blood	Bacterial meningitis and chronic renal failure
YMC2011/8/B10311	M	73	Blood	Malignant neoplasm of body of pancreas
YMC2011/11/B1440	M	81	Blood	Disseminated intravascular coagulation
YMC2011/11/B7578	F	50	Blood	Esophageal varices with bleeding

Supplementary Table 2: Total RNA-sequencing read counts and normalized values for *OmpK35*, *OmpK36*, and *OmpA* for four carbapenem-resistant *K. pneumoniae* isolates using the reads per kilobase per million mapped reads (RPKM), relative log expression (RLE), and trimmed mean of M-value (TMM) normalization methods

Strain	Total read count			RPKM (CV = 0.342)			RLE (CV = 0.3396)			TMM (CV = 0.3387)		
	OmpK35	OmpK36	OmpA	OmpK35	OmpK36	OmpA	OmpK35	OmpK36	OmpA	OmpK35	OmpK36	OmpA
**YMC2014/1/R777**	310	8883	80301	160.44	3058.34	28946.41	695.61	13762.02	124406.28	787.42	15578.33	140825.8
**YMC2014/3/P345**	409	102	97682	99.12	29.52	29599.14	481.65	148.87	142571.01	528.96	163.5	156576.51
**YMC2014/4/B5656**	660	47890	37797	165.92	8964.75	7407.97	773.52	43376.97	34235.11	864.92	48502.29	38280.25
**YMC2014/5/U865**	827	89186	154300	135.72	8312.48	15057.33	698.23	44385.23	76790.54	771.49	49042.43	84847.93

CV, coefficient of variation

Supplementary Table 3: Protein bands from SDS-PAGE identified using an Agilent 6530 Accurate-Mass Q-TOF instrument

Strain (band no.)	SDS-PAGE band size	Match to	Identified protein	Score	Nominal mass (Da)	Calculated pI	Sequence coverage	Peptide score	Peptide sequences (remaining sequences for each band in Supplementary File 2)
YMC2014/1/R777 (1)	35.5 kDa	gi407808361	OmpA	1648	37055	5.52	96%	38	K.GSVDNGAFK.A
								56	K.GSVDNGAFKAQGVQLTAK.L
								42	K.LGYPITDDLDIYTR.L
								53	R.LGGMVWR.A
(2)	37 kDa	gi221192796	OmpK36	719	39907	4.60	76%	37	K.LDLYGK.I
								43	K.FGDAGSFDYGR.N
								37	K.NGSVSGEGALSPTNNGR.T
								48	R.LGDQNSKLALGR.G
								63	K.YDANNIYLATQYTQTYNATR.A
YMC2014/3/P345 (3)	35.5 kDa	gi407808361	OmpA	1359	37055	5.52	96%	35	R.MAYKGSVDNGAFKAQGVQLTAK.L
								52	K.GSVDNGAFKAQGVQLTAK.L
								42	K.LGYPITDDLDIYTR.L
								46	R.LGGMVWR.A
YMC2014/4/B5656 (4)	35.5 kDa	gi407808361	OmpA	1340	37055	5.52	96%	37	R.MAYKGSVDNGAFKAQGVQLTAK.L
								57	K.GSVDNGAFKAQGVQLTAK.L
								51	K.LGYPITDDLDIYTR.L
								85	R.SEHDTGVSPVFAGGVEWAVTR.D
(5)	37 kDa	gi221192796	OmpK36	931	39907	4.60	84%	34	K.LDLYGK.I
								51	K.FGDAGSFDYGR.N
								47	K.NGSVSGEGALSPTNNGR.T
								57	R.AGSLGFANK.A
YMC2014/5/U865 (6)	35.5 kDa	gi407808361	OmpA	1552	37055	5.52	96%	38	K.DNTWYAGGK.L
								49	K.GSVDNGAFKAQGVQLTAK.L
								45	R.LGGMVWR.A
								55	R.SEHDTGVSPVFAGGVEWAVTR.D
(7)	37 kDa	gi221192796	OmpK36	779	39907	4.60	69%	37	R.LAFAGLK.F
								61	K.FGDAGSFDYGR.N
								42	K.NGSVSGEGALSPTNNGR.T
								64	R.AGSLGFANK.A
ATCC 13883 (8)	35.5 kDa	gi7442975	OmpA	1565	38135	6.00	75%	40	K.DNTWYAGGK.L
								57	K.AQGVQLTAK.L
								61	K.LGYPITDDLDIYTR.L
								55	R.ADSKGNYASTGVSR.S
(9)	37 kDa	gi295881600	OmpK36	1304	40791	4.61	75%	47	R.VGVKGETQINDQLTGYGQWEYNVQANNTESSSDQAWTR.L
								37	R.LAFAGLK.F
								60	K.FGDAGSFDYGR.N
								53	R.NYGVVYDVTSWTDVLPEFGGDTYGSDNFLQSR.A
(10)	37.1 kDa	gi3650340	OmpK35	690	39486	4.66	55%	53	K.MVGEHVWTTNGDTSSDDTTYAR.I
								35	R.LAFAGLK.A
			+					64	K.AGEYGSFDYGR.N
								49	K.FNQLDDNDYTK.A
		gi555025728	OmpK36	627	40747	4.61	46%	35	R.LAFAGLK.F
								62	K.FGDAGSFDYGR.N
								43	R.ANGVATYR.N
								42	K.INLLDENDFTR.R
